# Novel US-CpHMD Protocol to Study the Protonation-Dependent Mechanism of the ATP/ADP Carrier

**DOI:** 10.1021/acs.jcim.2c00233

**Published:** 2022-04-20

**Authors:** Nuno F. B. Oliveira, Miguel Machuqueiro

**Affiliations:** BioISI—Biosystems & Integrative Sciences Institute, Faculty of Sciences, University of Lisboa, Campo Grande, C8 bdg, 1749-016 Lisboa, Portugal

## Abstract

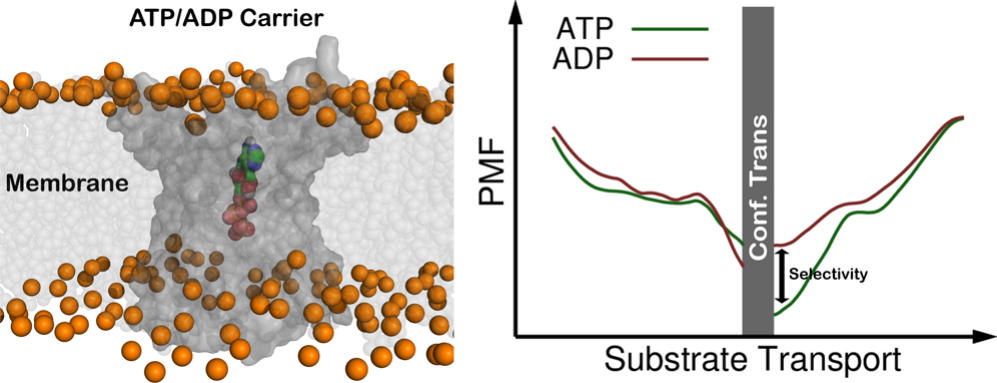

We have designed a protocol combining constant-pH molecular dynamics (CpHMD) simulations with an umbrella sampling (US) scheme (US-CpHMD) to study the mechanism of ADP/ATP transport (import and export) by their inner mitochondrial membrane carrier protein [ADP/ATP carrier (AAC)]. The US scheme helped overcome the limitations of sampling the slow kinetics involved in these substrates’ transport, while CpHMD simulations provided an unprecedented realism by correctly capturing the associated protonation changes. The import of anionic substrates along the mitochondrial membrane has a strong energetic disadvantage due to a smaller substrate concentration and an unfavorable membrane potential. These limitations may have created an evolutionary pressure on AAC to develop specific features benefiting the import of ADP. In our work, the potential of mean force profiles showed a clear selectivity in the import of ADP compared to ATP, while in the export, no selectivity was observed. We also observed that AAC sequestered both substrates at longer distances in the import compared to the export process. Furthermore, only in the import process do we observe transient protonation of both substrates when going through the AAC cavity, which is an important advantage to counteract the unfavorable mitochondrial membrane potential. Finally, we observed a substrate-induced disruption of the matrix salt-bridge network, which can promote the conformational transition (from the C- to M-state) required to complete the import process. This work unraveled several important structural features where the complex electrostatic interactions were pivotal to interpreting the protein function and illustrated the potential of applying the US-CpHMD protocol to other transport processes involving membrane proteins.

## Introduction

Electrostatic forces are pivotal in most biomolecular processes, being the main driving force of molecular interactions due to their long-range influence.^1,2^ Often, these forces play a role in attracting substrates to binding sites^3^ and can also define the binding strength between two molecules because the shape complementarity is commonly enhanced by compatible electrostatic surfaces.^4^ Protein electrostatics can be attributed to the polar or charged groups in the amino acid side chains usually located at their surfaces, some of which are able to undergo (de)protonation events depending on the pH and environmental conditions. As a consequence, the protein electrostatic surface can often be modulated by the solution pH, leading to protonation events that can ionize or neutralize key amino acid side chains.^5−7^ These changes in the protein electrostatic surface at different pH values can be a powerful driving force for protein stability and function.^8−11^

From the large number of proteins found in a cell, transmembrane transporters are highly important due to their role in selectively transporting molecules across the biological membrane.^12^ This class of proteins is often involved in the binding and transport of compounds with varied chemical compositions, with special emphasis on charged molecules. Therefore, the electrostatic surface of a transmembrane transporter protein is crucial for substrate attraction, binding, and efficient diffusion through the protein channel cavity.^13,14^ Electrostatic interactions can also modulate the selectivity of the transporter when amino acid side chains with a given charge are presented in the channel opening, creating a filter that favors compounds with the opposite charge.^14−16^

Correctly describing the electrostatic forces involved in biomolecular processes is highly appealing due to their ubiquitous nature and strong impact; however, this is extremely difficult using conventional techniques. Such a challenge has prompted the development of new computational methods to model these phenomena in microscopic detail. Furthermore, the rapid increase in computational power has leveraged several advances in the field of computational structural biology, allowing the study of increasingly complex systems.^17−20^ Several methodologies have already been shown to effectively describe the electrostatic environments of biomolecular systems, including the constant-pH molecular dynamics (CpHMD) method, which allows for a correct description of pH effects in proteins,^21−44^ membranes,^45−50^ and protein/ligand systems.^51−53^ Although this technique increases the realism of our MD simulations by sampling the correct protonation equilibria in biomolecules at a given pH value, it is still limited by their conformational sampling restrictions. In order to circumvent these barriers, it is common to rely on enhanced sampling techniques, such as umbrella sampling (US),^54^ where a bias is introduced in the MD simulations to force sampling of high energy states. The correct ensemble properties can still be recovered by applying a re-weighting procedure.^54,55^ A combination of the potential bias with CpHMD simulations (US-CpHMD) provides a very powerful technique that should be able to address structural properties along the slow biomolecular process and still capture the pH effects on the electrostatic environment of our system.

The ATP/ADP carrier (AAC) is a transporter protein composed of six transmembrane helices and highly abundant in the inner mitochondrial membrane.^56−58^ This protein is responsible for the exchange of both ATP and ADP across the impermeable inner mitochondrial membrane. In vivo, ATP is the main provider of chemical energy and it is obtained from ADP in the mitochondrial matrix; therefore, the balance of these two molecules’ concentration is essential for the cell.^13,57−59^ Such as many other transporters, the AAC has two distinct conic shape conformations, ensuring that only one access to the cavity is available at each moment. The two distinct conformational states are the C-state, where the inner cavity is opened to the intermembrane space (cytoplasmic side), and the M-state, where the cavity is opened to the mitochondrial matrix.^59−61^ In a normal living cell, the C-state binds mostly ADP from the cytoplasm and guides it to the bottom of its cavity. Once there, a conformational transition will shift the protein into the M-state, allowing the ADP molecule to be released and diffuse into the mitochondrial matrix. While in the M-state, the AAC attracts the abundant ATP, triggering a similar process in reverse, where the conformational change to the C-state triggers ATP release into the cytoplasm/nuclei, where it is needed.

**Figure 1:**
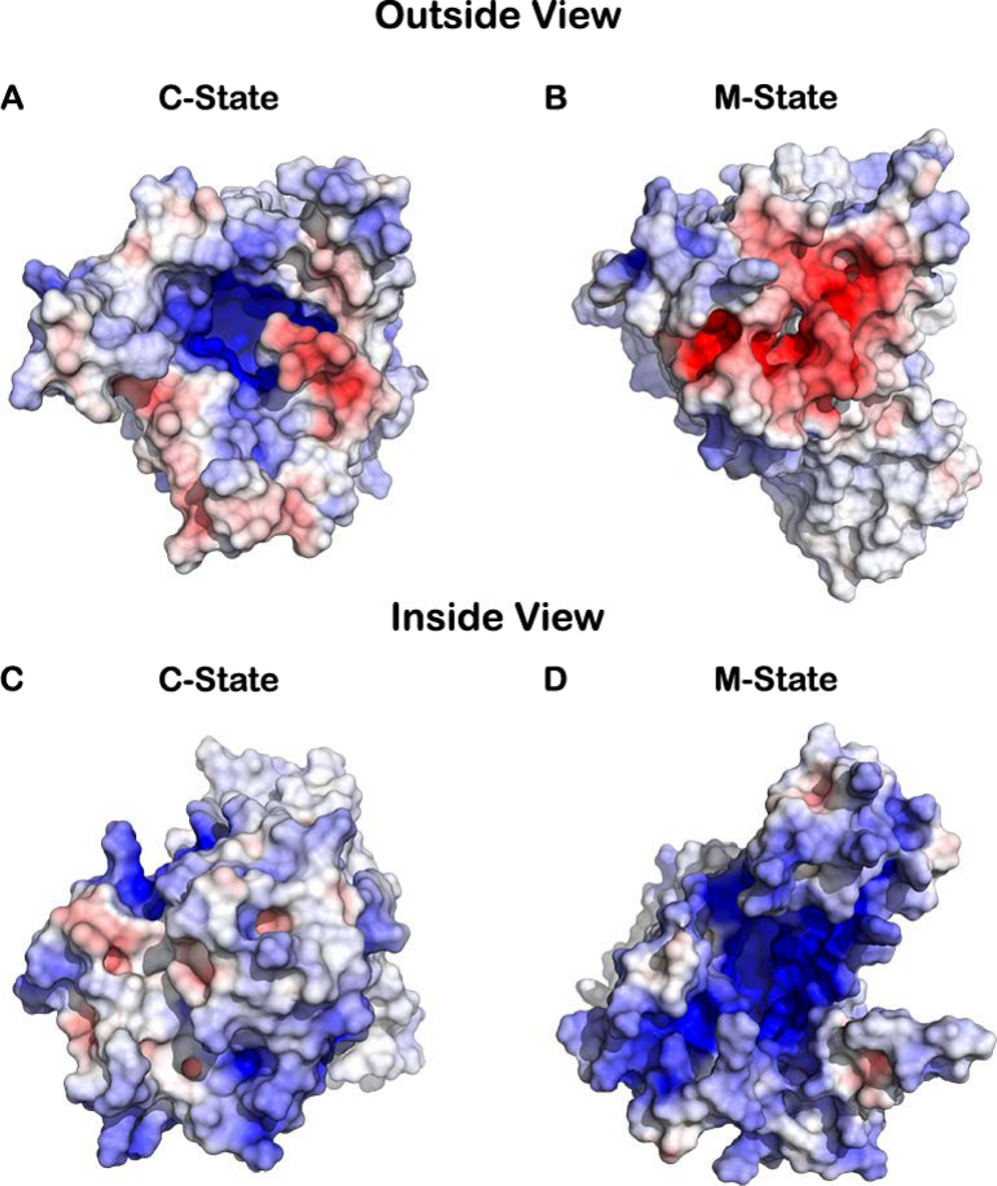
Electrostatic surface of the AAC in both the C-state (A,C) and the M-state (B,D), viewed from outside (A,B) and from inside the mitochondrial matrix (C,D). Figures were rendered using PyMOL^65^ and the electrostatic surface was calculated using APBS plugin (default parameters).^66^ The mitochondrial membrane was excluded from the APBS calculation and from visualization.

The high selectivity toward the negatively charged ADP/ATP indicates that strong electrostatic interactions between these molecules and the AAC positive inner cavity (Figure 1) are at play.^57,62−64^ This has been confirmed by mutating several charged residues present in the cavity, which results in several degrees of transport impairment.^67^ Additionally, electrostatic interactions are also involved in the stability of the apo AAC structure. In the C-state, a group of salt-bridge interactions is usually formed at the bottom of the cavity, known as the matrix salt-bridge network, which is responsible for closing the matrix side access to the cavity, giving stability to the conic shape of this state.^58,61^ This salt-bridge network is commonly observed in the mitochondrial carrier superfamily because it is formed by residues that lie in the conserved sequence motif present on the odd-numbered helices P-X-D/E-X-X-K/R-X-K/R-(20 amino acids)-D/E-G-X-X-X-X-W/Y/F-K/R-G.^59,63^ In the absence of crystal structures for the M-state, there have been a few bioinformatics studies suggesting the presence of a similar salt-bridge network in this state.^58,59,61,68^ They have found a highly conserved sequence motif present on the even-numbered helices, F/Y-D/E-X-X-K/R, that closes the access to the cavity from the cytoplasmic side, hence being called the cytoplasmic salt bridge.

In this work, we study the energetics and the structural details of the ADP/ATP transport along the AAC channel. To achieve this, we designed a new enhanced sampling protocol by combining the US scheme with our CpHMD method.^23,27,48,49^ This allowed capturing the correct conformational description of the transport process at the desired pH, including the protonation changes that are essential to adequately modeling the electrostatic forces along the channel.

## Methods

### Homology Modeling and System Setup

The setup of the C-state system started with the *Bos taurus* X-ray structure (PDB id: 1OKC(69)), which was cleaned and inserted into a pre-equilibrated 256 phosphatidylcholine (POPC) bilayer. Removing the AAC clashing lipids resulted in 200 POPC molecules (100 in each bilayer) solvated with ∼11,300 SPC water molecules.^70^ The mitochondrial membrane lipid composition is complex and can include anionic lipids (e.g., cardiolipin)^71^ whose charges can change with pH.^47^ To circumvent this level of complexity, we have approximated our system to pure POPC, which forms a stable lipid bilayer in the fluid phase.

The *B. taurus* M-state structure is yet to be resolved. However, a structure from the *Thermothelomyces thermophila* (PDB id: 6GCI) organism has recently been obtained,^61^ which is a good homology model to achieve the *B. taurus* counterpart. We used the MODELLER package^72^ for sequence alignment and obtained several *B. taurus* M-state structure models. Using the DOPE score, the best model was selected, and its Ramachandran plot^73^ was used to confirm its quality. An overlap of both template and model structures (Figure S1 of Supporting Information) was also made to visually inspect the selected construct. After confirming the quality of our model, we proceeded to insert it into a POPC bilayer, analogous to the C-state.

### Molecular Mechanics/MD Settings

Both AAC systems were simulated using molecular mechanics/MD (MM/MD) using GROMACS 2018.6 software^74,75^ and the GROMOS 54A7 force field.^76^ The v-rescale thermostat^77^ was used to maintain the system at 310 K with a coupling constant of 0.01 ps and the semi-isotropic pressure was kept at 1.0 bar using the Parrinello–Rahman barostat^78,79^ with a compressibility of 4.5 × 10^–5^ bar^–1^ and a coupling constant of 1.0 ps. Long-range electrostatic interactions were treated with the particle mesh Ewald (PME) approach using a Verlet scheme with a 1.0 nm single cutoff. The same cutoff was used to treat the van der Waals interactions.^80^ The bonds of protein and POPC lipids were constrained using p-LINCS,^81^ while water was constrained using the SETTLE algorithm.^82^

The systems were energy minimized using the steepest descent algorithm. A careful step-wise MD initialization procedure was applied to deal with the high energy interactions between proteins and lipids, which were not tackled by the minimization steps. This protocol consisted of four steps: (i) 50 ps MD with a 0.001 ps time step, generating velocities (*NVT*) for all the molecules but the AAC, which was kept frozen; (ii) 200 ps MD with 0.0001 ps time step using the Parrinello–Rahman barostat (*NPT*) and the freeze groups were replaced by position restraints of 10^4^ kJ mol^–1^ nm^–2^ applied in the backbone of the protein; (iii) in the 100 ps MD step run, we change the position restraint strength to 10^2^ kJ mol^–1^ nm^–2^ and the time step to 0.001; and (iv) in the final step, we run a 500 ps MD segment with a restraint strength of 10 kJ mol^–1^ nm^–2^ and a time step of 0.002. After the initialization protocol, three long MM/MD pre-equilibration runs (1 per replicate) were performed (500 ns) for both the C- and M-state systems. In these runs, the convergence of our membrane–protein system was evaluated following several equilibration properties, including the root-mean-square deviation (RMSD), secondary structure content, lipid patch area, and distance (*z*-axis) between the membrane center and the AAC cavity geometrical center (Figures S2, S3, S4, and S5 of Supporting Information).

### ATP and ADP Parameters and p*K*^mod^ Calibration Procedure

In order to study the complete transport process performed by the AAC, it is required to obtain accurate parameters for ATP and ADP. In the GROMOS 54A7 force field,^76^ there are already parameters for ATP with a proton in the last phosphate (−3*e*). Because we are interested in using both the protonated and deprotonated forms of ATP and ADP, this requires a parameterization of the fully ionized form of these substrates. In our work, we have avoided a complete parameterization of these species by starting from the already available ATP parameters. The charges of each intermediate phosphate unit were slightly adjusted to add up to −1*e*. For the final phosphate group in both molecules, we should consider the poorly solvated conditions present in the binding cavity, for which we have shown that classic parameterization methods, such as RESP,^83−85^ are inadequate.^53^ In that work, we curated a new charge set for both protonated and deprotonated terminal phosphate groups to correctly describe the desolvation effects, p*K*_a_ calculations, and interactions with the positively charged amino acids in the PLCγ1 SH2 domain.^53^ Because of the similarity between the pocket environments of the SH2 domain and our AAC protein, and because we are particularly interested in pH-dependent binding affinities, we adopted those curated ATP parameters. From ATP, building ADP only required the removal of a nonterminal phosphate unit (Table S1 and Figure S6 of Supporting Information).

In our CpHMD methodology, we require p*K*^mod^ values for ADP and ATP, which can easily be calibrated by running CpHMD simulations in water and adjusting these values in order to mimic their water p*K*_a_ values measured experimentally (6.3 and 6.5 for ADP and ATP, respectively^86^). We simulated these molecules in water (50 ns) with a starting p*K*^mod^ of zero and calculated the correct p*K*_a_ shifts (1.48 and 5.97 for ADP and ATP, respectively), which resulted in the final p*K*^mod^ values of 4.78 and 0.53 for ADP and ATP, respectively. The high p*K*_a_ shift observed for ATP results from the strong electrostatic interactions between the titrating site (the terminal phosphate group) and the neighboring phosphate groups that are always charged at pH 6.5. This shift decreases significantly for ADP (from 5.97 to 1.48) because there is only one phosphate unit next to the titrating site.

### CpHMD Settings

Prior to studying the substrate interaction with the AAC, we performed a full pH titration study of our transporter, aiming to capture and only select the most relevant residues titrating at the desired pH value. Three CpHMD replicates of 100 ns at pH values of 4.0, 5.0, 6.0, and 7.0 were performed for both apo-AAC systems and C- and M-states, allowing the titration of all aspartates, glutamates, histidines, tyrosines, cysteines, lysines, and both termini. These simulations were run using GROMACS 5.1.5^75^ and the GROMOS 54A7 force field.^76^ Because we are using PME, the system needs to be neutral, or the total charge will be neutralized by a background charge correction.^87^ In the CpHMD framework, protonation/deprotonation events are allowed, leading to fluctuations in the system total charge that, if not mitigated, can lead to significant artificial effects.^87^ In this work, we have circumvented this issue by adding a given fixed number of counter ions at each pH value to bring the charge fluctuations around neutrality. In this scenario, the background charge correction is only used to correct very small charge deviations from zero. Small 20 ns pre-runs were performed to estimate the total charge of our system at the different pH values. The appropriate number of Cl^–^ counter ions were added at each pH (Table S2 of Supporting Information). Upon the completion of the production simulations, 20 ns were discarded to ensure system equilibration. We have plotted titration curves and calculated p*K*_a_ values for all the titeratable residues by fitting the data on a Hill curve.

#### Poisson–Boltzmann and Monte Carlo Settings

All the Poisson–Boltzmann (PB) calculations were performed using Delphi v5.1 software.^88^ Atomic charges were obtained from the GROMOS 54A7 force field directly, while atom radii were derived from the Lennard-Jones parameters of each atom type.^89^ The dielectric constant values of 2 and 80 were used for the solute (AAC + membrane + ADP/ATP) and the solvent, respectively.^23,27^ A probe of 1.4 Å radius was used to generate the molecular surface. An ionic strength of 0.1 M was used throughout the simulations, with an ion exclusion layer of 2 Å. To perform the calculations over the entire system, including the lipid patch, a grid was applied in two steps. A large grid of ∼1 Å was first used, followed by a focusing grid of ∼0.25 Å. On the larger grid, the electrostatic potential was calculated using periodicity in the *x*/*y* plane,^47,84,85^ a convergence threshold of 0.01, and relaxation values of 0.20 and 0.75 in the linear and nonlinear iteration steps, respectively.

The final protonation states were sampled from the PB-derived free energies using Monte Carlo (MC) calculations with PETIT.^90,91^ Proton tautomerism was included in the calculations for all the titeratable residues. A total of 10^5^ MC cycles were performed, each of these cycles attempting to change individual and pairs of sites with interactions larger than 2 p*K* units, using the Metropolis^92^ criterion.

### Steered Molecular Dynamics

**Figure 2:**
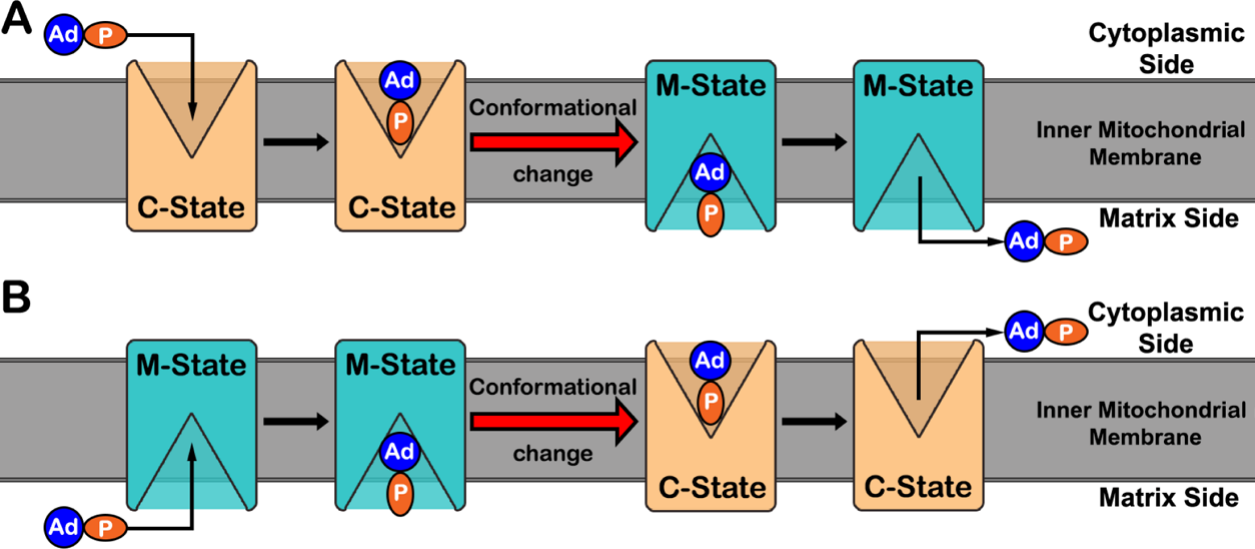
Mechanism scheme for the import (A) and export (B) processes. The inner mitochondrial membrane is represented by a dark gray slab and the AAC protein (rectangular shape) is exchanging between the C- and M-states, colored in orange and blue, respectively. The ADP and ATP substrates are represented by two oval shapes colored blue and orange to represent the adenosine and phosphate moieties, respectively.

A biasing potential US scheme was used to obtain an atomistic description of the complete import and export transport processes. As a reaction coordinate, we have selected the distance in the *z*-axis (membrane normal) between the terminal phosphate of the substrate and the center of our protein cavity. This was defined using a group of Cα atoms located in the central position of the channel in both states of the carrier (Figure S7 of Supporting Information). To accurately describe these processes, we need proper conformations of each state of the transport mechanism. This is particularly challenging because the AAC undergoes a major conformational transition during each process. Our MD simulation timescale is unable to capture such a large conformation change (from the C- to M-state for the import process and vice versa for the export), hence we will address the transport process without performing such a conformational transition. As a consequence, the transport will be described by combining umbrellas from two distinct segments the entry and exiting of substrates, which is marked by the change of the conformation state in our US protocol (Figure 2).

We used steered MD (sMD) to generate starting configurations for each US window that were compatible with the desired transport process. In this protocol, we apply a pulling bias potential to drag our substrates across the simulation box. When inside the channel, our substrates are not able to freely rotate and invert their initial orientation. In all our test runs, ADP and ATP insert their leading phosphate groups into the positively charged protein cavity. However, because after the conformational transition the substrate will keep its original orientation, we also need to generate initial configurations with the adenosine groups facing the AAC central cavity. We performed four sMD simulations starting with ATP at the bottom of the protein cavity in both conformational states and with either the phosphate (entry position) or the adenosine (exit position) facing the center (Figure S8 of Supporting Information). In these systems, ATP was pulled to the outside using the pull group (phosphate or adenine) that was facing the protein exterior. Furthermore, four extra sMD simulations were performed where ATP was pulled in the opposite direction without a conformational transition and through the closed portion of the channel (Figure S9 of Supporting Information). The umbrella windows starting from these closed conformations will provide information on the energy barrier that would happen without the conformational transition and help identify the substrate position where the conformational transition needs to take place. All the sMD were performed using the ATP molecule because ADP can be easily obtained by removing a phosphate unit. It should be noted that the strategy to start the sMD runs from the channel center to the outside of the protein has the advantage of avoiding non-optimal pathways that could lead to major protein/membrane deformations.

In all the sMD runs, we pulled ATP until it reached the end of the simulation box, usually ranging from ∼0 (defined by the AAC center; see Figure S7 of Supporting Information) to ∼− 4 or ∼+ 4 nm, depending on the direction. The pull force was set as an umbrella type with a “direction” geometry with a force constant of 1000 kJ mol^–1^ nm^–2^ and a rate of 0.025 nm ns^–1^. From the trajectories, we extracted representative conformations at reference *z*-axis positions of each US window.

### Coupling US with CpHMD

In a US scheme, we start by dividing our system into several windows and assigning each a proper starting conformation. To model the complete import and export processes, we need to generate US windows in the *z*-axis ranging from −4 to +4 nm, where the zero reference is at the center of the protein. Inside the channel, we created US windows at 0.1 or 0.2 nm intervals and applied a high biasing force constant of 1000 kJ mol^–1^ nm^–2^. On the more solvent-exposed regions, to decrease the computational cost, we have reduced the number of US windows and their force constant values to 500 or 250 kJ mol^–1^ nm^–2^ (Table S3 of Supporting Information). The decision to add more or less spaced windows was based on the sampling quality, which was assessed by visually evaluating the US window overlap. Furthermore, in most solvent-exposed umbrellas (<−2.0 and >+2.0 nm) the substrate regains its rotational freedom, therefore, we can re-use the same umbrellas for either scheme (import and export processes).

In the two schemes modeled (import and export), we start from US windows performed with one conformational state and, at some point, corresponding to the conformational transition, we change to the other state. To pinpoint the appropriate window where this conformational change in the AAC should occur, we computed the potential of mean force (PMF) energy without this change. From these PMF profiles, we observe clear minima at the cavity center (∼+0.4 and ∼+0.0 nm for the C- and M-states, respectively), followed by a significant energy barrier due to the closed portion of the channel (Figure S10 of Supporting Information). Therefore, we adopted the +0.2 nm US window as the position where the conformational change is imposed. This large conformational change also leads to a discontinuity in the PMF calculations, which was addressed by calculating two independent energy profiles, starting from the water phase (zero energy setting).

We performed 150 ns CpHMD simulations at pH 7 for each US window for ADP and ATP in both transport schemes. A shorter list of residues was allowed to titrate in the US-CpHMD and the selection criteria were based on the proximity to the channel and their protonation sensitivity at pH 7 (Figure S11 of Supporting Information). The final list included only Asp, Glu, and His residues. Lys22 and 32 were not included because their depressed p*K*_a_ values,^67^ due to desolvation and electrostatic interactions, were often larger than 7. The presence of a negatively charged substrate will stabilize the protonated form of lysines, which should significantly increase their p*K*_a_ values. The first 20 ns of each umbrella CpHMD simulation were discarded to ensure good system equilibration.

### Simulation Analyses

The analyses performed throughout this work used the tools available in the GROMACS package^75^ or developed in-house. All the graphics were created using gnuplot^93^ and PyMOL.^65^ In the apo-AAC CpHMD simulations, the presented errors were calculated from the three replicated standard errors of the mean. The PMF profiles were calculated using the weighted histogram analysis method (WHAM),^55^ available in the GROMACS 5.1.5 package tools.^75^ The bootstrap error calculations performed using the WHAM implementation in GROMACS lead to significantly small error values and do not capture correctly the conformational variability observed in our CpHMD simulations. To improve the error estimation of our PMF profiles, we split the CpHMD simulations into two time segments and calculated the PMF profile in each. The energy difference between the two segments in each position provides a better estimation of the PMF error values. To obtain the protonation profiles and minimum residue distances along the transport coordinate, a re-weighting protocol was applied to remove the bias inserted in each US window. For each umbrella, the errors presented were calculated using the standard error of the mean coupled with an autocorrelation function to determine the number of independent blocks (correlation value of 0.1) in the simulations.^20^

## Results and Discussion

### Apo-AAC CpHMD Simulations

We performed CpHMD simulations of the apo-AAC in both conformational states and obtained titration curves (and p*K*_a_ values) for the residues titrating in the ∼3 to ∼8 pH range (Table S4 of Supporting Information). Larger differences in p*K*_a_ values between protein and water^94^ are most likely correlated with the site surrounding environment and can help pinpoint important electrostatic interactions or desolvation effects. In a membrane transporter, desolvation effects can be mainly felt either inside the protein channel or at the water membrane interface. Asp10 is one of these cases where the high p*K*_a_ values can easily be attributed to the effect of the surrounding lipids (Figure S12 of Supporting Information). Lys22 and 32 are part of the binding site and the matrix salt-bridge network,^59^ respectively, and present decreased p*K*_a_ values, probably due to a combination between desolvation and direct interactions with other positively charged residues abundant in the pocket (Figure S13 of Supporting Information). These p*K*_a_ shifts are in qualitative agreement with those in the literature (6.0–6.5 range).^67^ However, these residues are required to be charged at physiological pH, with only a noticeable decrease in activity at values above 7.5.^67,95^ Therefore, p*K*_a_ values in the 7–8 range are probably in better agreement with the AAC pH activity profile. It should be noted that these depressed p*K*_a_ values for Lys22 and 32 completely disappear in the presence of a substrate.^67^

**Figure 3:**
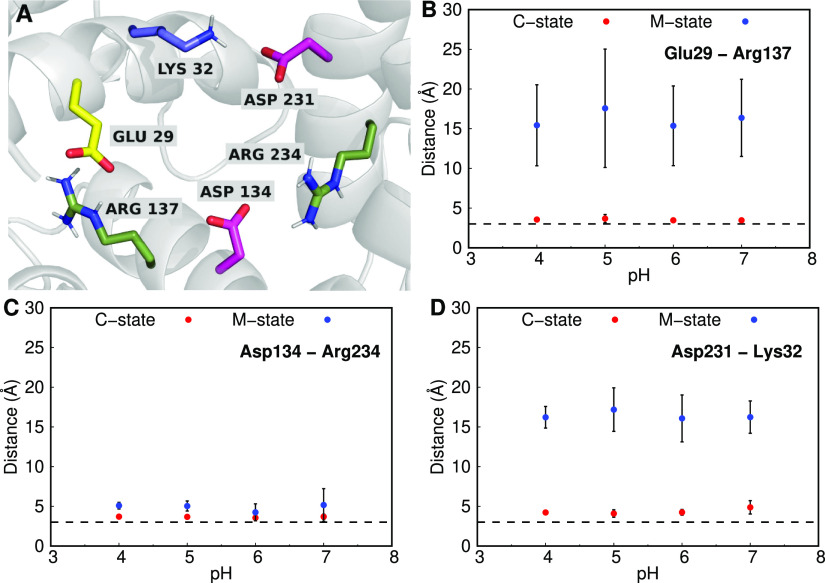
Structural representation of the matrix salt-bridge network (A) and the average distances between the residue pairs involved (B–D). The AAC is shown as a gray-colored cartoon, with the key Asp, Glu, Lys, and Arg residues shown as sticks colored (carbon atoms) in magenta, yellow, forest green, and slate blue, respectively. The average distances between the salt bridge pairs are shown for all the pH values and for the C- (red) and M-states (blue) of the AAC. A dashed line at 3 Å was added to illustrate a typical stable salt bridge distance.

We also observed a significant decrease in the p*K*_a_ values of residues Glu29, Asp134, and Asp231, in the C-state conformation of the AAC (Table S4 of Supporting Information). These residues are located at the cavity bottom, which should result in increased p*K*_a_ values due to desolvation. However, the strong interactions with positively charged residues were able to overcome this effect, shifting the p*K*_a_ values in the opposite direction. In fact, these three residues have been identified in the literature to be a part of the matrix salt-bridge network,^59,63^ which is formed by some elements of the conserved sequence of the mitochondrial carrier superfamily, P-X-D/E-X-X-R/K-(20 amino acids)-D/E-G-X-X-X-X-W/Y/F-K/R-G (Figures 3A and S14 of Supporting Information). The matrix salt-bridge network is important for the AAC because it stabilizes the closed cavity from the matrix side of the membrane when in the C-state. The average distances between these acidic residues and their cationic partners in the carrier pocket (Figure 3B–D) confirmed the presence of strong salt-bridge interactions between Glu29 and Arg137, Asp134 and Arg234, and Asp231 and Lys32. As expected, shifting the protein from the C- to M-state leads to either some destabilization (Asp134–Arg234) or even the complete separation of the salt-bridge residues involved (Glu29–Arg137 and Asp231–Lys32). The disruption of this network is probably an important step in the conformational change that is coupled with the transport process.

### US-Constant-pH Molecular Dynamics

The complete transport mechanism performed by the AAC is quite complex and involves several steps, including the substrate movement, the large conformational transition, and the protonation changes coupled to the transient, but strong, electrostatic interactions. The description of this process in atomistic detail is almost impossible experimentally and is highly challenging using computational techniques due to its slow timescale. To overcome these limitations, we devised an enhanced sampling protocol based on US coupled with CpHMD simulations. With this approach, we capture the conformational space and changes in protonation states (at pH 7.0) of our system where the substrate molecules cross the AAC protein.

**Figure 4:**
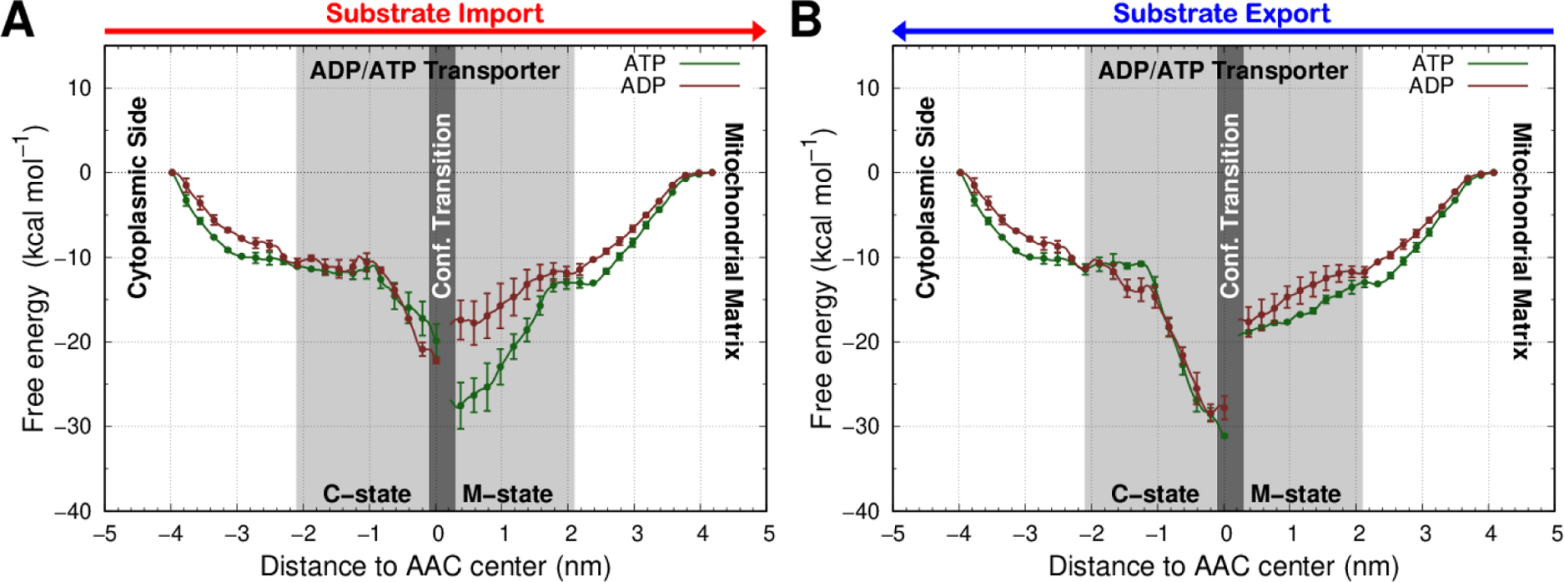
PMF energy profiles of both substrates, ATP (brown) and ADP (green), in the import (A) and export (B) transport processes. The red and blue arrows, for import and export, respectively, were included at the top to identify the direction of the substrate transport. The light gray region delimits the positions inserted in the AAC channel. The umbrellas using the AAC C- and M-states are identified with specific labels and separated by a dark gray region marking the major conformational transition. The PMF error bars, which are very small, are shown only every five points for clarity.

After confirming a good overlap between the US windows selected in our protocol (Figure S15 of Supporting Information), we calculated the energetic profile (PMF) associated with the substrates crossing in either direction (Figure 4). In both the import and export processes, the free energy of our substrates acquires a negative slope when approaching the AAC cavity. This can be explained by the strong attractive forces that both the C- and M-states exert over the substrate molecules, which helps to guide and orient them to the cavity. This attraction is due to the electrostatic interactions between the positive electrostatic funnel that is present in the AAC cavity and the negatively charged phosphate groups of both adenosine nucleotides. After reaching the bottom of the channel, for both processes and both substrates, a large conformational transition (between the C- and M-states) is required to occur. Because this transition cannot be captured in our US protocol, there are discontinuities in the PMF calculations at the bottom of the channel (dark gray region in Figure 4). Therefore, the difference in PMF energy values pre- and post-transition can be mainly attributed to the specific conformational change. In the import process, after the transition to the M-state, there is a significantly lower energy barrier (∼10 kcal mol^–1^) for the exiting pathway of the ADP molecule when compared to ATP (Figure 4A). This pronounced difference induced by the C- to M-state transition selectively favors the import of ADP over ATP. In the export PMF, the ADP and ATP profiles behave similarly, indicating the absence of selectivity in this transport (Figure 4B).

These results should be interpreted in the correct physiological context. In the import process, the ADP molecules are captured by the C-state AAC with the cavity open to the cytoplasmic side of the membrane. This has two major difficulties. First, the substrate is transported against the membrane potential of the inner mitochondrial membrane. Second, the large volume of the outside compartment, approximately the cytoplasmic volume due to the high permeability of the outer mitochondrial membrane, is several times larger than the matrix. This leads to a lower substrate concentration/availability on the cytoplasmic side compared with the matrix side. These factors probably resulted in an evolutionary pressure on the AAC to be highly efficient in capturing the substrates from the cytoplasm side and to generate some type of selectivity toward ADP, which is the desired substrate of the import process. In contrast, the most important export process in vivo deals with the transport of ATP molecules from the matrix, where they are highly concentrated, to the cytoplasm, where they are needed. This takes advantage of both the concentration gradient and the mitochondrial membrane potential, which probably resulted in a weaker evolutionary pressure on the AAC to acquire more efficiency or selectivity.

It should be noted that despite the presence of selectivity toward ADP in the import process, the PMF profile differences between both molecules, under the right conditions, should not prevent the transport of the undesired molecule. This is in line with experiments where reversing the membrane potential leads to inverted substrate transport in vitro.^57,62,96,97^ Furthermore, a similar mechanism is found for the ATP synthase protein with an abolished membrane potential. This leads to an inversion of the normal process and the protein becomes an ATPase, exporting protons from the matrix and hydrolyzing ATP.^98,99^

**Figure 5:**
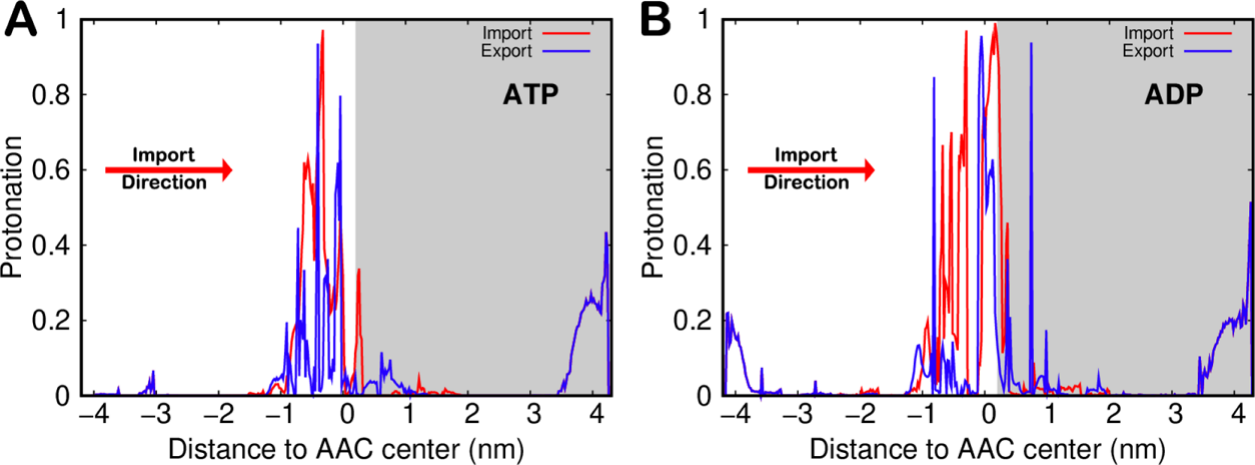
Protonation profiles of ATP (A) and ADP (B) for the import (red lines) and export (blue lines) processes obtained from the US-CpHMD simulations. White and gray backgrounds were used for umbrellas simulated in the AAC C- and M-states, respectively. In the more solvated regions, away from the AAC and the membrane, both import (red) and export (blue) lines are superimposed because the same umbrella windows were used.

An important advantage of performing US-CpHMD simulations is the ability to sample the protonation states of several important residues along with the transport processes. A very good example is the protonation profile of the substrate terminal phosphate group at pH 7.0 (Figure 5). Considering that the bulk p*K*_a_ values of ATP and ADP are 6.5 and 6.3, respectively,^86^ we expect ∼20% protonation at the simulated pH. Indeed, this is observed in umbrella windows located at longer distances, where the substrate titration curves are not perturbed by the AAC. At shorter distances between umbrellas, the substrates interact with the transmembrane protein cationic cavity, which favors their deprotonated/charged state. Interestingly, in the ATP umbrellas in the C-state conformation, a 4 nm distance (from the AAC center) was not enough to fully recover its bulk protonation, indicating a strong interaction between the cationic cavity and this longer substrate.

When crossing the AAC cavity, we note a significant tendency to protonate the terminal phosphate group of both substrates in the import process. This is most likely due to the desolvation effect, which is more pronounced in the −1 to +1 nm range. However, the cationic/anionic residue ratio is higher in the pocket located on the M-state side, which counteracts the desolvation effects and keeps a low average protonation of the terminal phosphate group. The protonation profiles in the export process show that very little protonation is required compared with the import process. This difference can also be related to the evolutionary pressure exerted on these processes. There is a major energetic advantage to protonating anionic substrates when importing them into the mitochondrial matrix against the membrane potential. Of course, protonating these substrate molecules in the export process would be a significant disadvantage.

**Figure 6:**
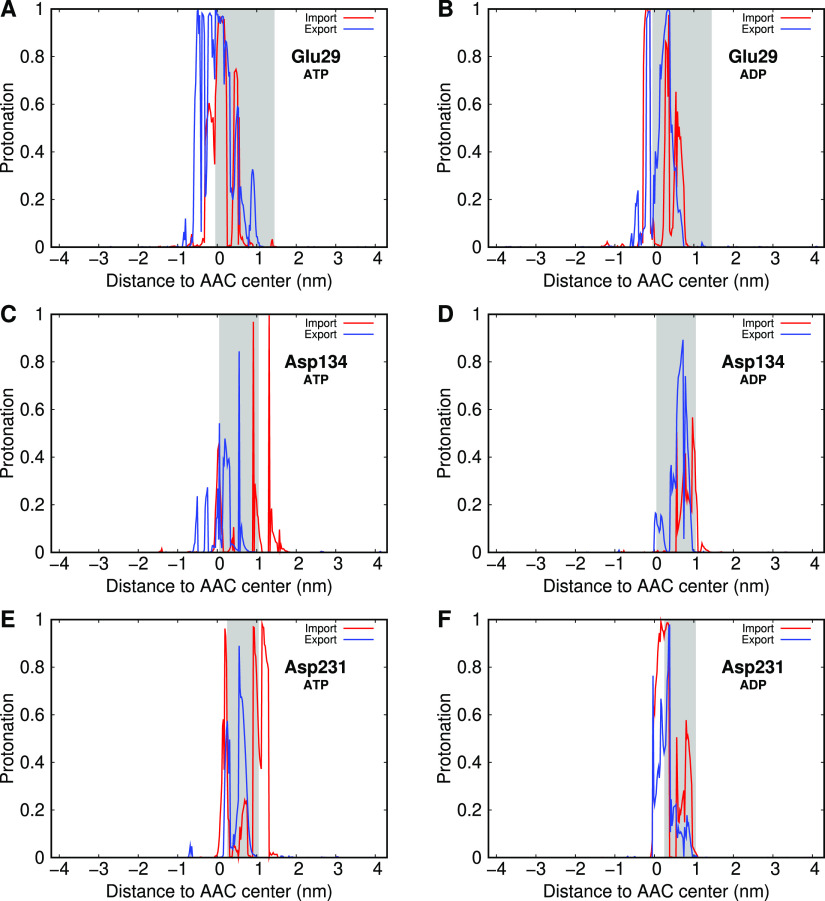
Protonation profiles of Glu29 (A,B), Asp134 (C,D), and Asp231 (E,F), during the transport of both ATP (A,C,E) and ADP (B,D,F) in the import process (blue line) and in the export process (red line). A gray-shaded area highlights the region sampled by each amino acid residue during the simulations, corresponding with the substrate interaction region.

In a similar analysis, we extracted protonation profiles for several residues in the AAC cavity during the transport processes in order to evaluate their direct interactions with the substrates. From these, we identified Glu29, Asp134, and Asp231 as important residues in the AAC pocket that modulate their protonation state upon interaction with the substrate (Figure 6). These are the key acidic residues involved in the matrix salt-bridge network, previously studied in the apo-AAC CpHMD simulations. Their profiles show an increase in protonation due to the proximity of the negatively charged substrates (gray shaded regions). These three residues are involved in salt-bridge interactions at the bottom of the C-state cavity, and their protonation will disrupt the matrix salt-bridge network. Because this network is stabilizing the C-state, its disruption may be the event that triggers the import conformational transition from the C- to M-state.

In the export process, there are also some salt-bridge interactions that stabilize the M-state. Bioinformatics studies have revealed a possible salt-bridge network on the F/Y-D/E-X-X-K/R conserved sequence motif.^59,61^ However, unlike the matrix counterpart, the cytoplasmic salt-bridge network is located at the AAC outer rim (Figure S16 of Supporting Information). Therefore, the residues involved in this network are less sensitive to the presence of the substrate in the M-state cavity. This is clear in the protonation profiles of residues Asp92 and Asp291, members of the cytoplasmic salt-bridge network, which do not protonate significantly in the M-state umbrellas (distances > 0 nm) (Figure S17 of Supporting Information). Only in the C-state and after the salt-bridge disruption do we observe some protonation of these residues (more clear for Asp92) due to the direct interaction with the negatively charged substrates. The lack of evolutionary pressure in the export process may have also contributed to the absence of a clear effect from the substrate present in the M- to C-state conformational transition.

## Conclusions

In this work, we developed an effective computational protocol, combining CpHMD simulations with a US scheme, which allowed us to study, in atomistic detail, the complex electrostatics of the AAC transport mechanism. In apo-AAC, we showed that the p*K*_a_ values of Lys22 and Lys32, located at the bottom of the cavity, were significantly shifted toward the physiological pH. We also identified several relevant p*K*_a_ downshifts on the three acidic residues that are known to be involved in the matrix salt-bridge network (Glu29, Asp134, and Asp231).

We coupled the CpHMD methodology with the US scheme to mimic the full transport process of ADP and ATP by the AAC protein. The import of anionic substrates along the inner mitochondrial membrane has a strong energetic disadvantage due to a smaller substrate concentration and an unfavorable membrane potential. These factors and the biological context have led to an evolutionary pressure on the AAC to develop several features that counteract those limitations. The results presented in this work have identified some of these key features. We observed that in the import process, the C-state is able to sequester substrates at longer distances when compared to the M-state in the export process. The transport energetic profiles (PMFs) revealed a clear selectivity in the import of ADP compared to ATP, while in the export, no selectivity was observed. The substrate protonation profiles revealed a tendency for both substrates to transiently protonate when being imported through the AAC cavity. This protonation step represents an important advantage when importing these substrates to the mitochondrial matrix against the membrane potential. Finally, we have observed a substrate-induced shift in the protonation of the three key acidic residues of the matrix salt-bridge network. This leads to the disruption of this network and suggests an active role in promoting the conformational transition required to complete the import process. In contrast, the export process is energetically favored, which resulted in no evolutionary pressure to improve the efficiency and selectivity of the AAC protein.

In summary, our model design, coupled with the US-CpHMD protocol developed for this work, allowed us to simulate the mechanism of ADP/ATP transport by the AAC with unprecedented realism. The CpHMD simulations allowed us to sample the correct protonation states at pH 7.0, while the US protocol helped us with the conformational/configurational sampling by overcoming the kinetic barriers in the transport process. It should be noted that studying the atomic details of such large and complex systems, such as a membrane carrier, is very challenging. Nevertheless, our simulations unraveled several important structural features where the complex electrostatic interactions were pivotal in interpreting the protein function.

## Data and Software Availability

The GROMACS package is freely available software used to perform MD simulations and can be downloaded at https://manual.gromacs.org/documentation/5.1.5/download. PyMOL v2.0 is also free software for molecular visualization and generating high quality images. It can be downloaded from https://pymol.org/2. As Supporting Information, we provide the code to run the CpHMD simulations, including the modified GROMOS 54A7 force field, which was extended to support the ADP and ATP titrating molecules. The system’s starting configurations and topologies are also included in the same file.

## Supporting Information Available

Structure alignment in homology modeling. RMSD and helix content of the AAC, area (*x*/*y*) of the system, distance of the AAC center to the membrane center in MD simulations, phosphate group charge sets used in this work for both substrates and visual aid to place the atom names average total charge of the system and number of counter ions added, structural representation of the Cα atoms used to define the AAC center, p*K*_a_ values for several residues of the AAC, starting configurations of the sMD runs, scheme for the transport processes without conformational transition, PMF energy profiles of substrate transport without conformational transition, structural representation of the titrating residues in US simulations, force constant assignment in US windows, structural representation of Asp10, Lys22, Lys32, and the MCF sequence motif, US distance histogram distributions, structural representation of the cytoplasmic salt-bridge network in the M-state, and protonation profiles of residues involved in the cytoplasmic salt-bridge network (PDF)
US-CpHMD code, force field files, and system structure and topology files (ZIP)
