# Supplementary material for: Novel US-CpHMD Protocol to Study the Protonation-Dependent Mechanism of the ATP/ADP Carrier
Source: J Chem Inf Model. 2022 Apr 20;62(10):2550–60. doi: 10.1021/acs.jcim.2c00233 (PMC9775199; doi:10.1021/acs.jcim.2c00233)
Supplement: Supplementary file 1 — ci2c00233_si_001.pdf [file ci2c00233_si_001.pdf]

Supporting Information

A novel US-CpHMD protocol to study the protonation-dependent mechanism of the ATP/ADP carrier

Nuno F. B. Oliveira and Miguel Machuqueiro*

BioISI - Biosystems & Integrative Sciences Institute, Faculty of Sciences, University of Lisboa, Campo Grande, C8 bdg, 1749-016 Lisboa, Portugal

E-mail: machuque@ciencias.ulisboa.pt

Phone: +351-21-7500112

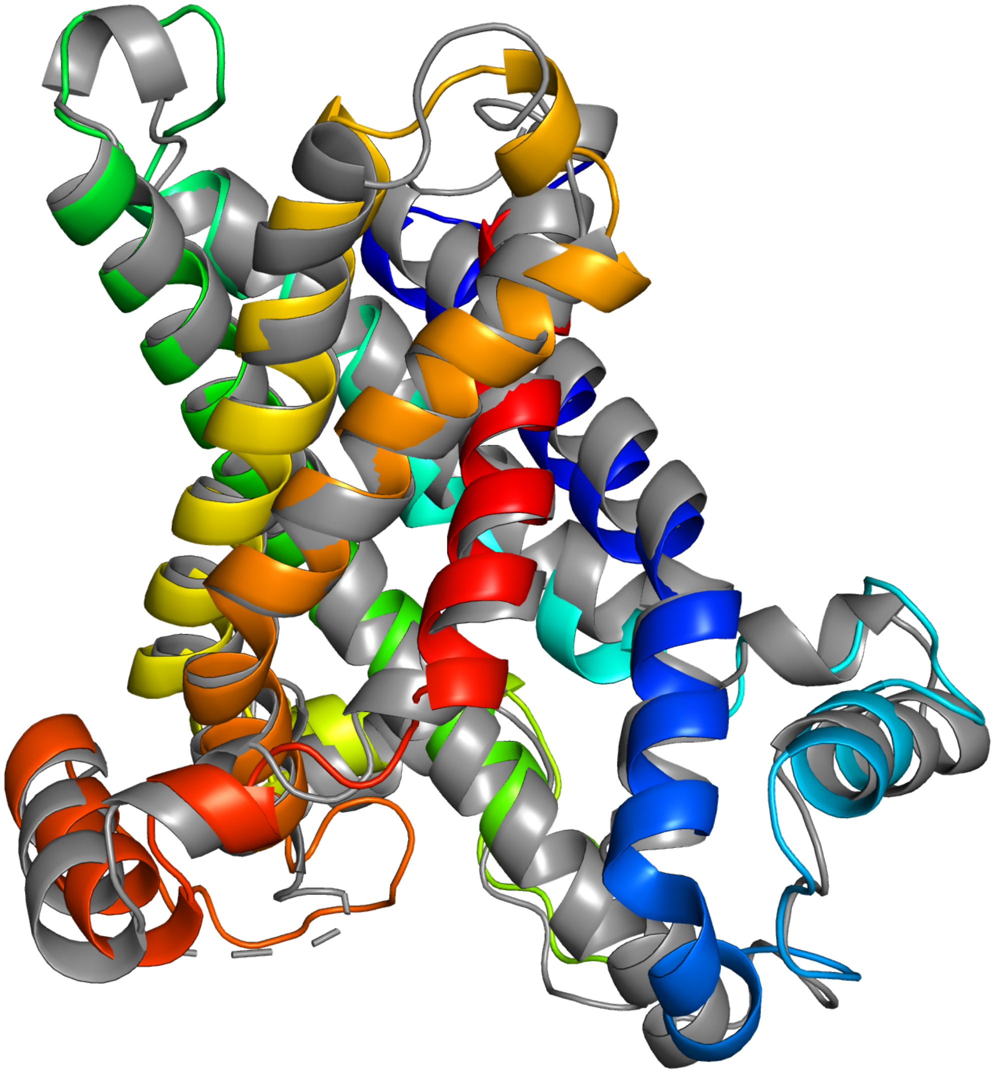

Figure S1: Structure alignment between AAC M-state template structure from *Thermothelomyces thermophila* (colored in gray) and the *Bos Taurus* M-state model obtained from homology modelling (colored with PyMOL Cα rainbow coloring).

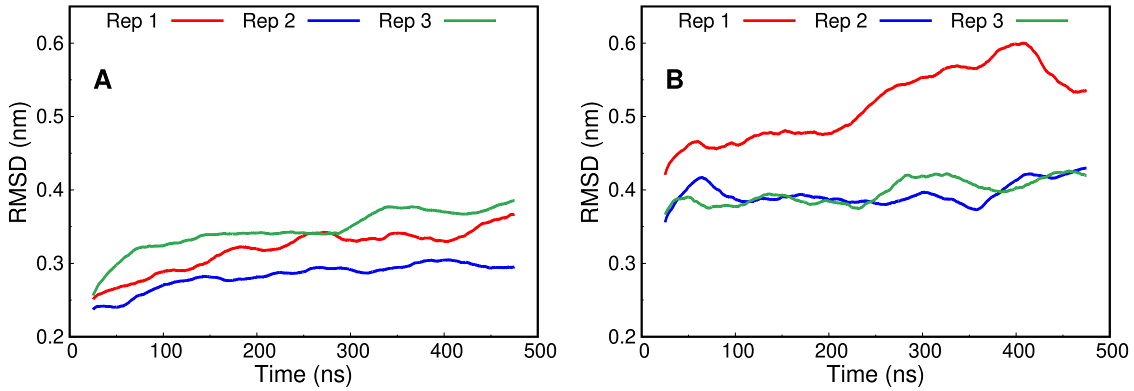

Figure S2: RMSD of the long MD runs. Each step is structurally compared with the structure obtained in the initialization procedure of the respective replicate. The RMSD of each replicate in its respective AAC state, C-state (A) and M-state (B), was plotted using gnuplot[1] and a floating window of 50 ns was used in order to reduce local fluctuations of each replicate curve. Replicate 1 of the M-state showed a pronounced deviation from the initial structure due to a transient loss of helical structure (see below Figure S3) in the region of residues 53-73 (h1-2 matrix α-helix), which showed to be highly flexible. This conformational rearrangement should not impact the transport process, since the segment involved is far away from the AAC channel.

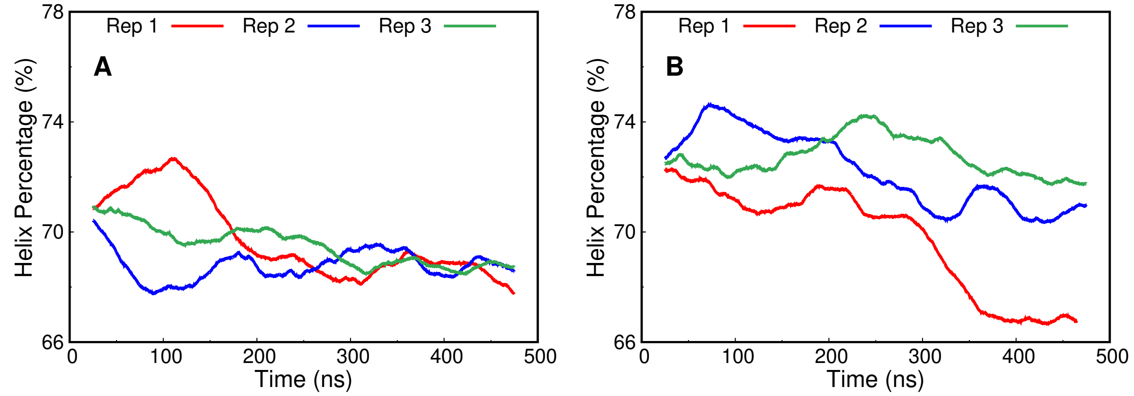

Figure S3: Percentage of helix content of the C-state (A) and M-state (B) during the MD runs, obtained using DSSP calculations.[2,3] On both plots a floating window of 50 ns was used in order to reduce local fluctuations.

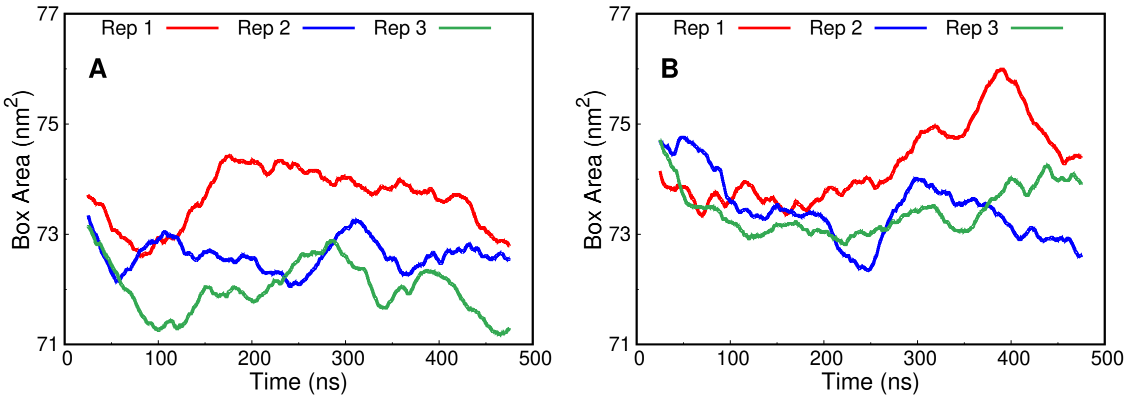

Figure S4: Area of the simulation system in the xy axis over time. Both C-state (A) and M-state (B) have each individual replicate plotted with a floating window of 50 ns in order to reduce local fluctuations.

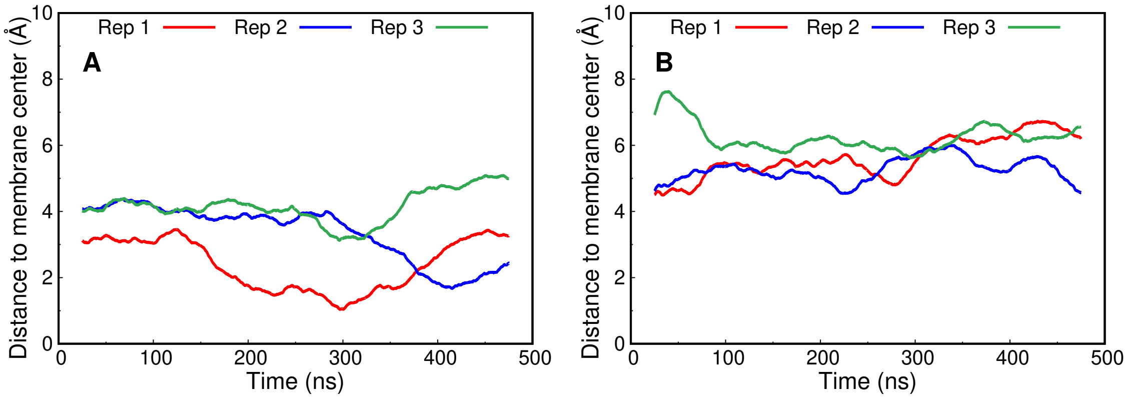

Figure S5: Distance to membrane center of the AAC cavity geometrical center in the C- (A) and the M-state (B). The distance values were plotted using a floating window of 50 ns in order to reduce local fluctuations.

Table S1: The phosphate group charge sets used in this work for both substrates, ADP and ATP, in protonated and deprotonated states. The original ATP charge set from the GROMOS 54a7 force field[4] was adopted and corrected in the terminal phosphate charges to provide a better description of the binding phenomena.[5] The ADP was build from ATP by removing one of the two non-terminal phosphate units, maintaining the remaining charge rationale. The atomic partial charges (in e units) are shown and the name correspondence to the structure can be found in Figure S6.

Forcefield	G54A7	This Work				
Atom	ATP	ATP	ATP+H	Atom	ADP	ADP+H
O5S	-0.360	-0.375	-0.375	O5S	-0.375	-0.375
PA	0.705	0.705	0.705	PA	0.705	0.705
O1A	-0.635	-0.665	-0.665	O1A	-0.665	-0.665
O2A	-0.635	-0.665	-0.665	O2A	-0.665	-0.665
O3A	-0.360	-0.375	-0.375	O3A	-0.050	-0.050
PB	0.705	0.705	0.705	PB	0.870	0.870
O1B	-0.635	-0.665	-0.665	O1B	-0.870	-0.310
O2B	-0.635	-0.665	-0.665	H1B	0.000	0.230
O3B	-0.360	-0.050	-0.050	O2B	-0.870	-0.870
PG	0.630	0.870	0.870	O3B	0.870	0.870
O1G	-0.548	-0.870	-0.310	—	—	—
H1G	0.398	0.000	0.230	—	—	—
O2G	-0.635	-0.870	-0.870	—	—	—
O3G	-0.635	-0.870	-0.870	—	—	—

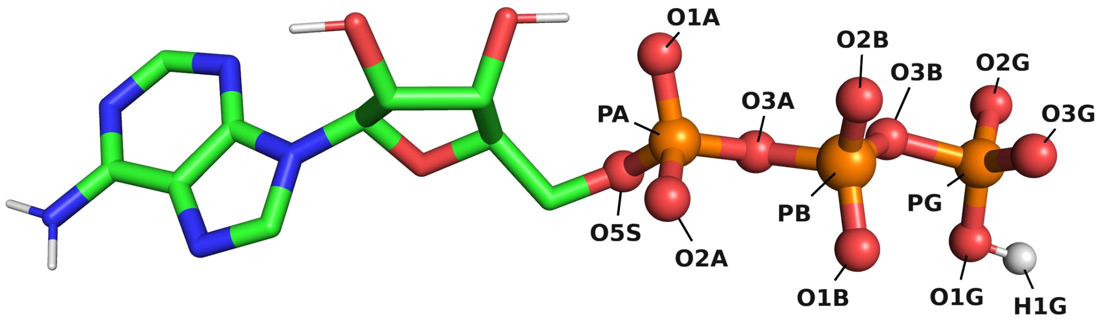

Figure S6: ATP structure highlighting the phosphate groups atom nomenclature used in this work.

Table S2: Average total charge and number of counter-ions added to the CpHMD simulations of the apo-AAC system in both C-state and M-state at the four different pH values (4, 5, 6 and 7).

pH	Total Charge		Counter Ions
	C-state	M-state	(Cl^-)
7	+17	+18	17
6	+20	+20	20
5	+23	+25	24
4	+30	+32	30

A B

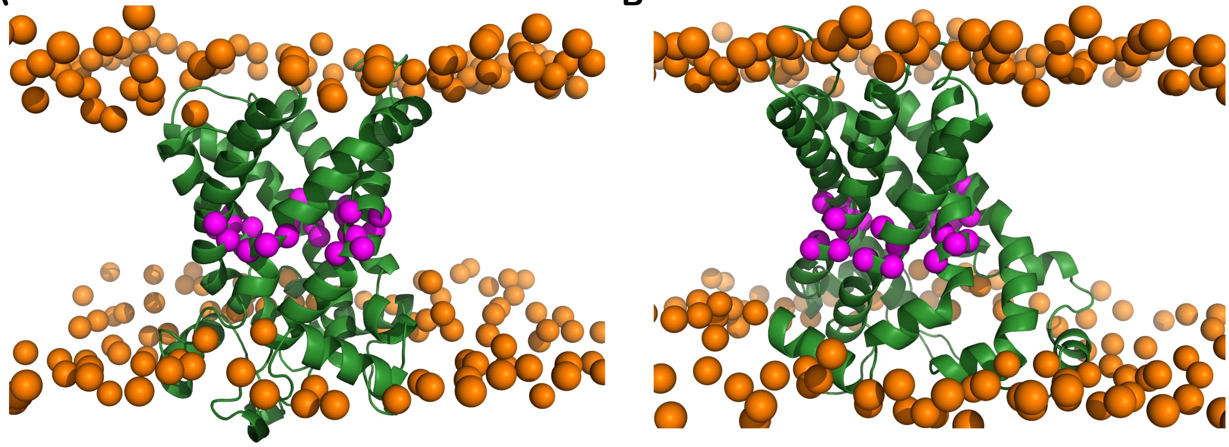

Figure S7: Structural representation of the $C\alpha$ atoms of the C- (A) and M-state (B) of AAC, selected to define the protein center. We used the following residues: 18, 21, 76, 77, 78, 123, 125, 126, 128, 129, 179, 180, 181, 182, 184, 222, 223, 224, 225, 226, 275, 276, 277, and 278. The $C\alpha$ atoms are colored in magenta and represented as spheres, the AAC structure is shown as a cartoon and colored in forest green and the phosphorus atoms of the lipids are represented as orange spheres to identify the membrane region.

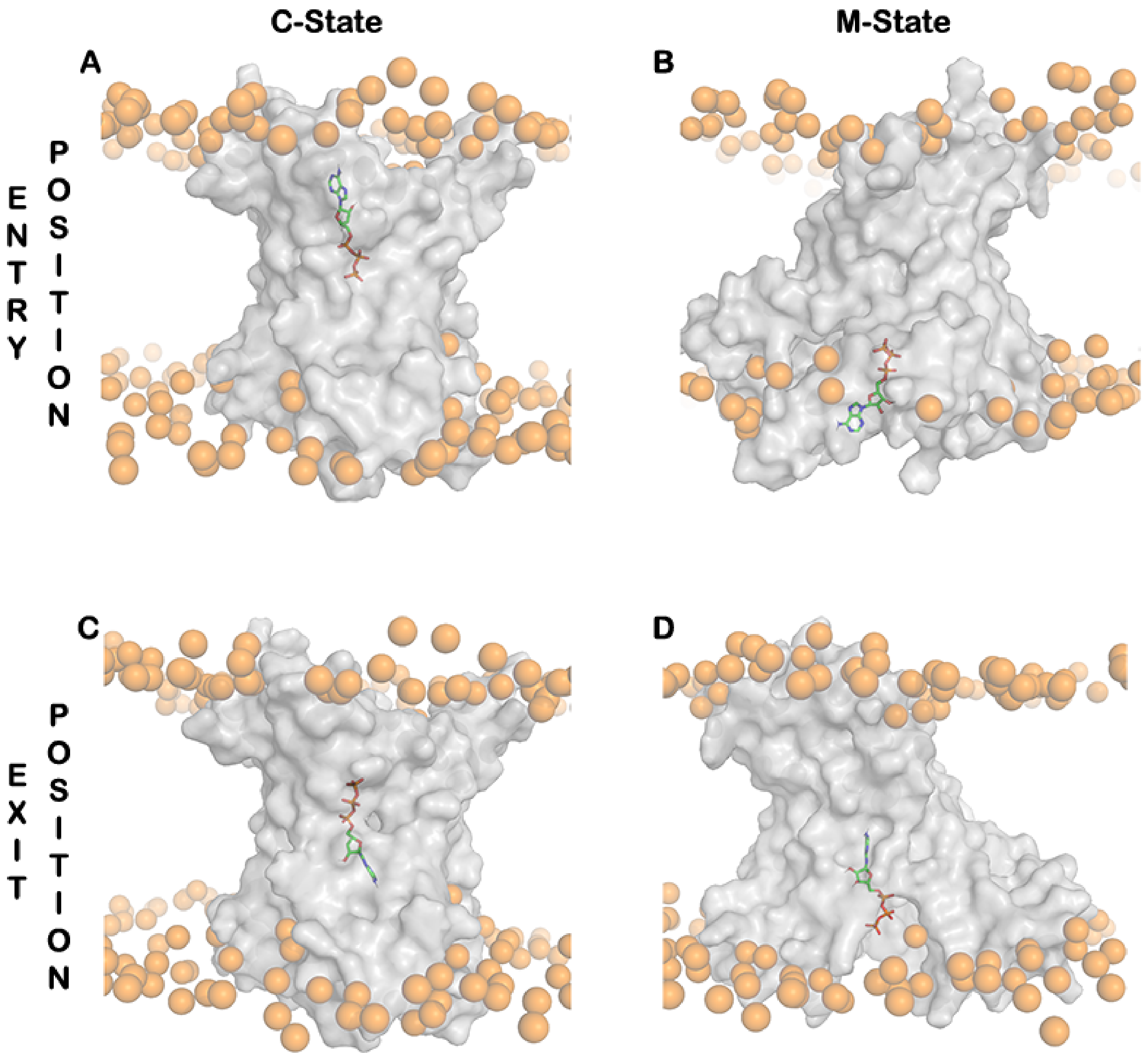

Figure S8: Starting configurations of each Steered MD run. Panels A and B represent AAC in the C- and M-states, respectively, with the substrate in entry position (pointing the phosphate group to the bottom of the cavity). panels C and D show the C- and M-state of AAC, respectively, with the substrate in exiting position (having the adenine group pointing to the bottom of the cavity). AAC is depicted as a grey transparent surface and the lipids phosphorus atoms are represented as orange spheres.

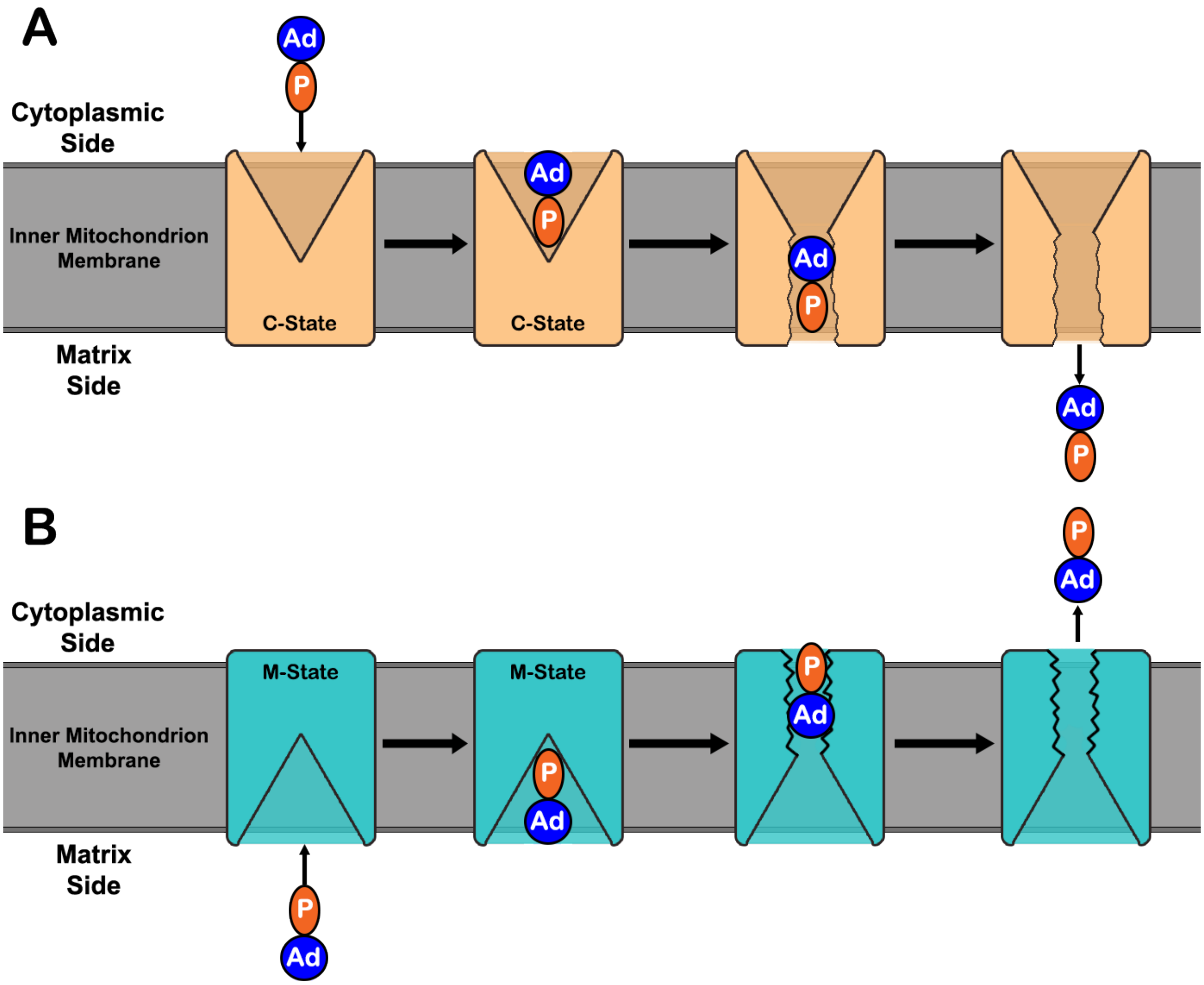

Figure S9: Scheme for the import (A) and export (B) processes without conformational transition. The inner mitochondrial membrane is represented by a dark grey slab and the AAC protein (rectangular shape) is exchanging between the C- and the M-state, colored in orange and blue, respectively. The ADP and ATP substrates are represented by two oval shapes colored blue and orange to represent the adenosine and phosphate moieties, respectively.

Table S3: Force constant values assignment to each US window along the substrate transport vector. At the terminal windows, a value of 500 kJ mol^{-1} nm^{-2} was used for ATP to avoid it reaching the edge of the simulation box.

**Position (nm)**	**Force constant (kJ mol^{-1} nm^{-2})**
-3.9	250 or 500
-3.7	500
-3.5	250
-3.2	250
-3.0	500
-2.7	500
-2.5	500
-2.4	1000
-2.2	1000
-2.0	1000
-1.9	1000
-1.8	1000
-1.6	1000
-1.4	1000
-1.2	1000
-1.1	1000
all windows spaced at 0.1 intervals	1000
+0.2	1000
all windows spaced at 0.2 intervals	1000
+3.0	1000
+3.3	500
+3.6	250
+3.9	250 or 500

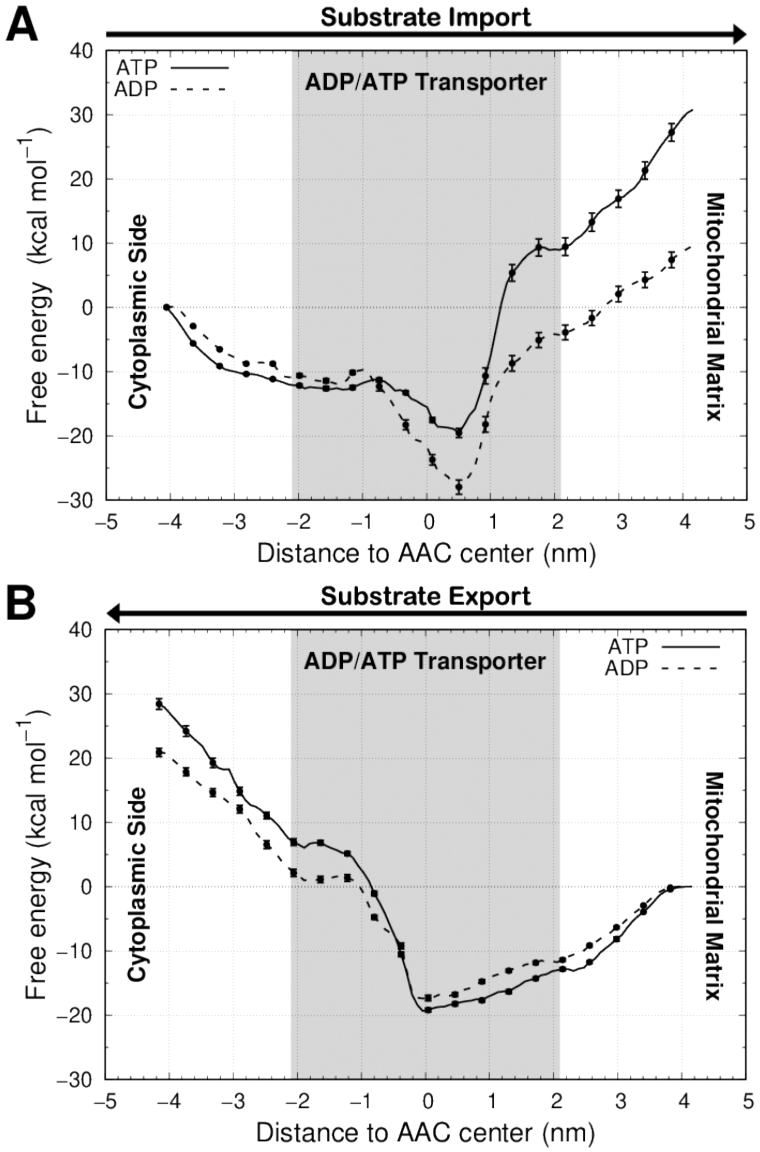

Figure S10: PMF energy profiles of both substrates, ATP (full line) and ADP (dashed line), in the import (A) and export (B) transport processes without conformational transition. Arrows have been included on top of the figure to point the direction the substrates are moving during the transport. A gray background area was used to represent the positions occupied by the AAC. The PMF error bars are shown only every 5 points for clarity.

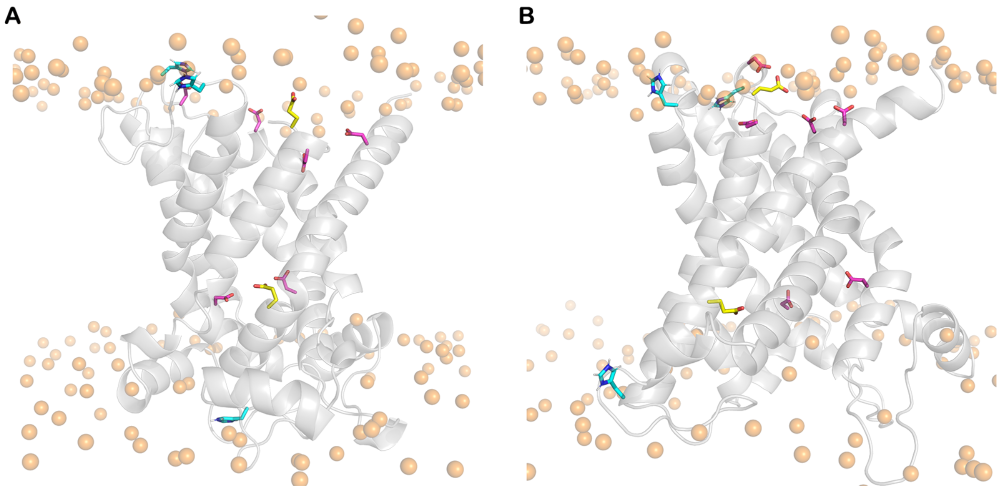

Figure S11: Structural representation of the C- (A) and the M-state (B) AAC. The titrable residues are shown in sticks. Aspartate residues (10, 92, 103, 134, 231, 291) are shown in yellow, glutamate residues (29, 292) are shown in pink and histidine residues (39, 105,208) are shown in cyan.

Table S4: pK_a values for several residues present on the AAC in both the C- and M-state. The errors reported were obtained from the standard error given by gnuplot[1] when fitting our data to a Hill curve. Values for the typical water exposed pK_a values of each residue (Water) were obtained from Reference.[6]

**Residue**		**Water**	**C-state**	**M-state**
**Asp**	2	**3.8**	3.4 ± 0.5	3.4 ± 1.3
	10		6.3 ± 0.1	>8
	55		4.3 ± 0.1	4.3 ± 0.1
	92		4.0 ± 0.1	5.4 ± 0.1
	103		3.1 ± 0.5	≈ 3
	134		<3	4.0 ± 0.3
	143		3.7 ± 3.5	3.9 ± 0.1
	167		4.5 ± 0.1	4.6 ± 0.1
	195		4.8 ± 0.3	4.4 ± 0.2
	203		3.9 ± 0.3	3.9 ± 0.1
	231		<3	3.1 ± 0.7
	247		3.8 ± 0.3	3.1 ± 0.6
	255		3.5 ± 0.2	4.2 ± 0.1
	263		3.9 ± 0.1	3.6 ± 0.0
	291		5.4 ± 0.3	4.1 ± 0.2
**Glu**	29	**4.3**	3.0 ± 0.7	3.2 ± 0.1
	47		4.0 ± 0.2	4.2 ± 0.2
	63		3.5 ± 0.1	5.7 ± 0.2
	152		4.1 ± 0.1	4.2 ± 0.1
	264		4.3 ± 0.1	4.3 ± 0.1
	292		4.8 ± 0.0	5.5 ± 0.3
**His**	39	**6.6**	5.3 ± 0.1	4.8 ± 0.4
	105		6.0 ± 0.1	5.8 ± 0.1
	208		5.3 ± 0.2	5.5 ± 0.1
**Lys**	22	**10.4**	7.9 ± 1.9	8.0 ± 0.7
	32		7.0 ± 0.3	>8

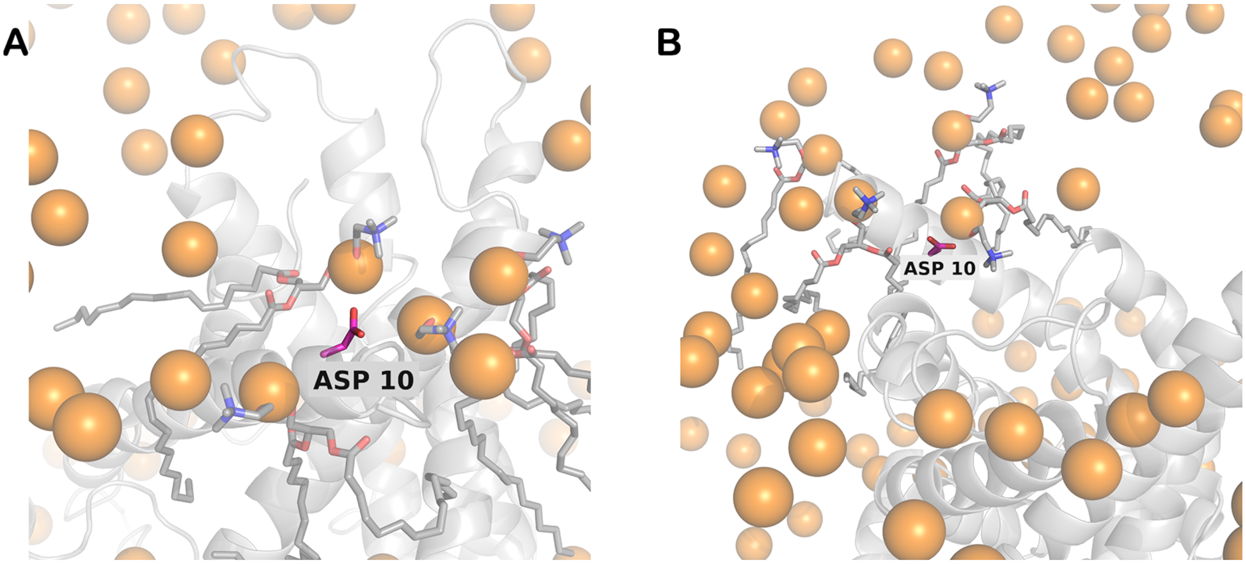

Figure S12: Structural representation highlighting the location of Asp10 in AAC M- (A) and C-states (B). Asp10 residue is shown in sticks with carbon atoms colored in magenta. AAC is shown as a cartoon and colored gray. The phosphorous atoms of the POPC lipids are shown as orange spheres. The lipids in the vicinity of the residue are shown with sticks and with carbons colored in gray.

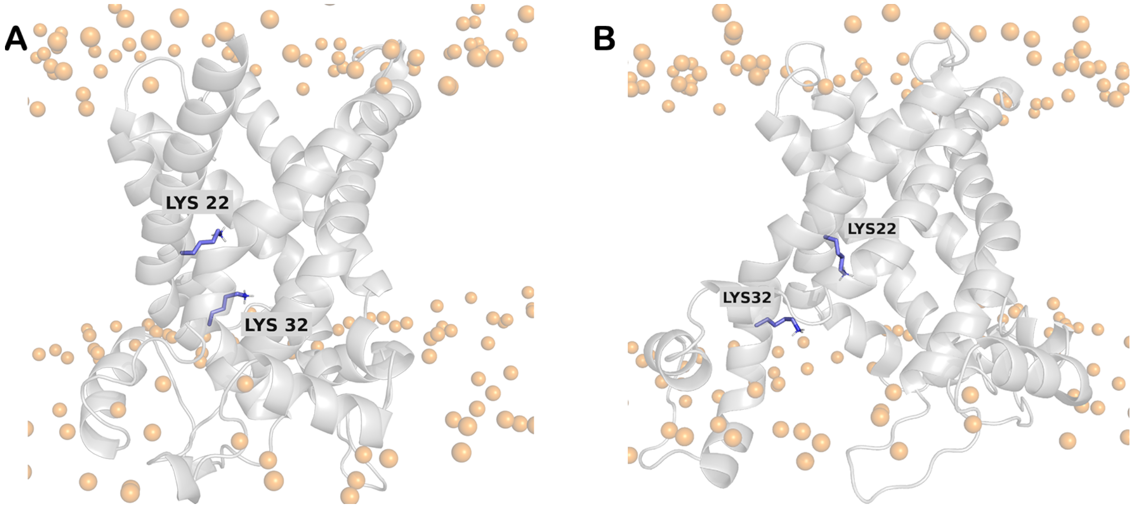

Figure S13: Structural representation highlighting the location of Lys22 and 32 in AAC M- (A) and C-states (B). Lysine residues are shown in sticks with carbon atoms colored slate blue. AAC is shown as a cartoon and colored gray. The phosphorous atoms of the POPC lipids are shown as orange spheres.

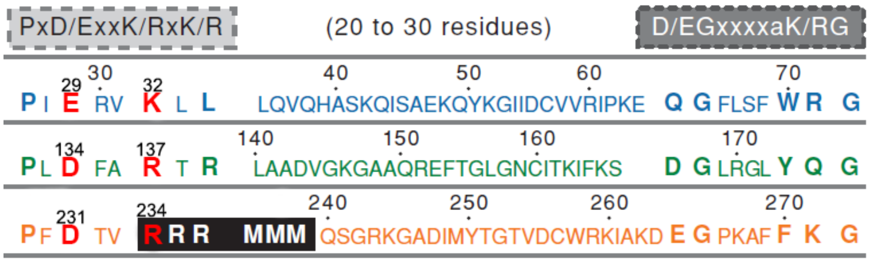

Figure S14: *Bos Taurus* MCF sequence motif (P-X-D/E-X-X-R/K-(20 to 30 residues)-D/E-G-X-X-X-X-W/Y/F-K/R-G) present in each odd-numbered trans-membrane helix (H1, H3 and H5). The AAC specific sequence domain (R-R-R-M-M-M) is highlighted in black. The residues, both acid and base, involved in the matrix salt-bridge network are marked in red. Adapted from.[7]

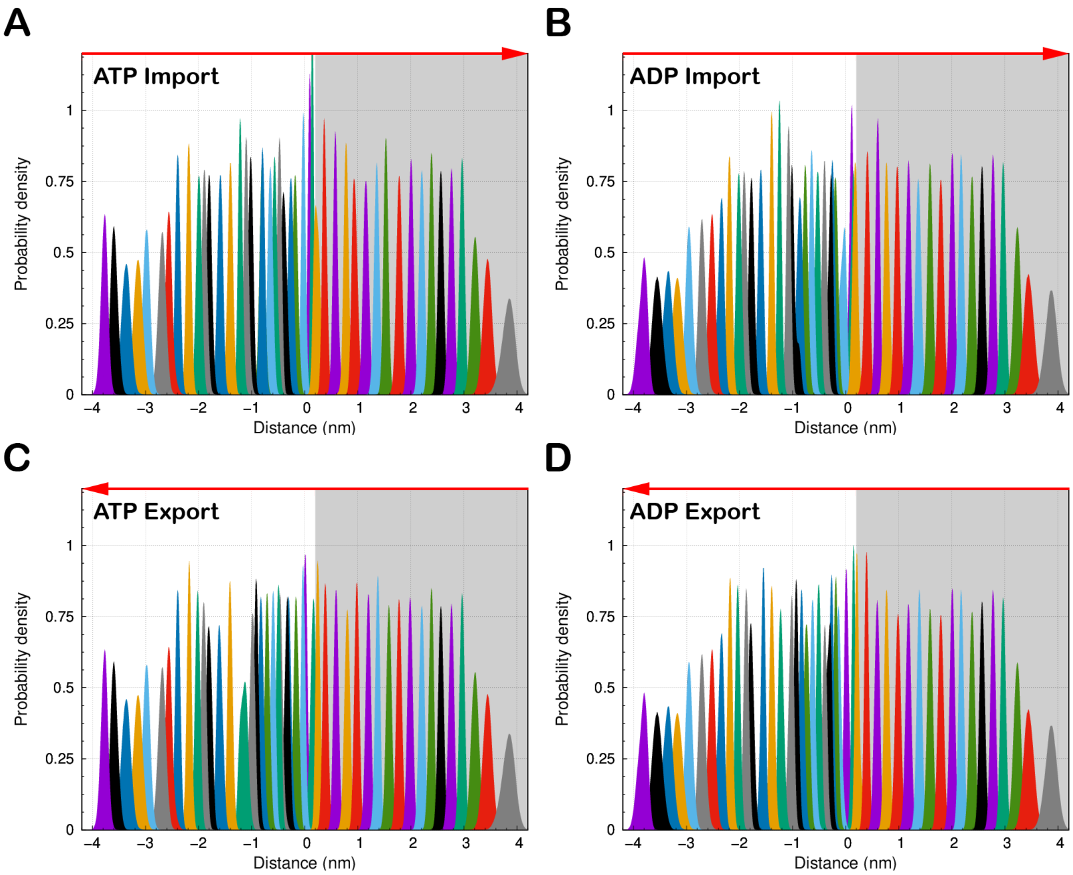

Figure S15: US distance histogram distributions for the import process of ATP (A) and ADP (B), and also the export of ATP (C) and ADP (D).

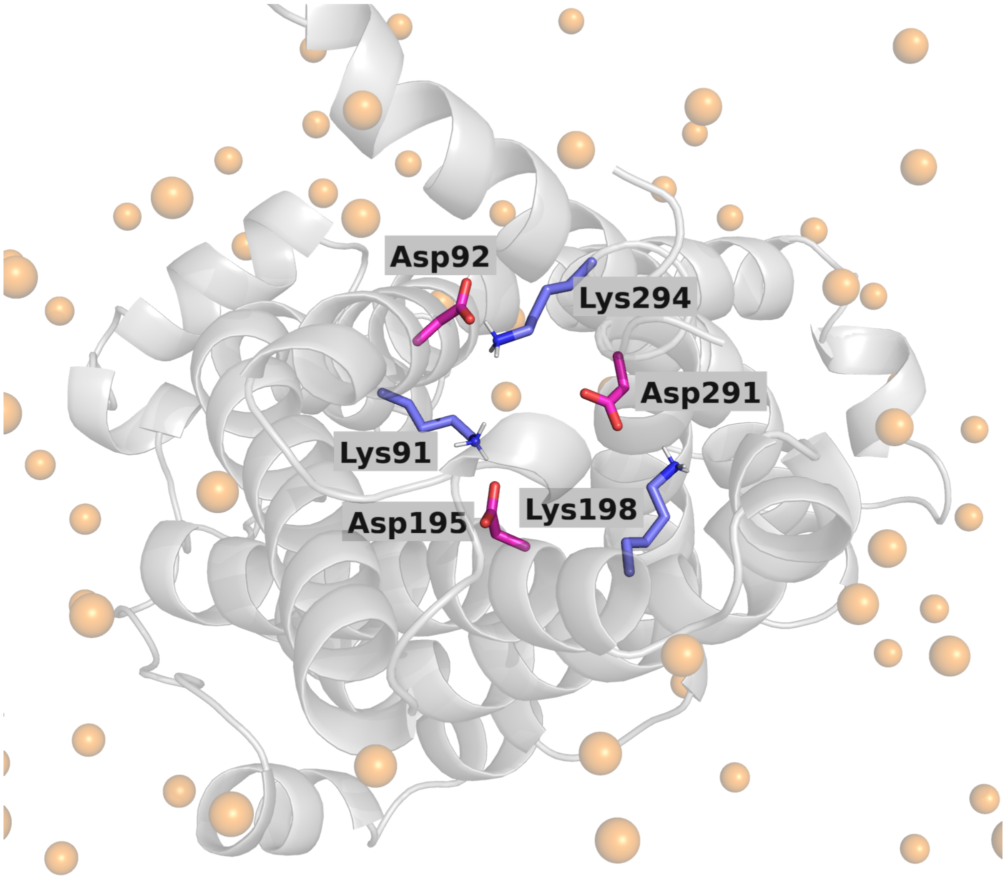

Figure S16: Structural representation of the cytoplasmic salt-bridge network in the M-state. AAC is shown as grey colored cartoon, with the key Asp, and Lys residues shown in sticks and colored (carbon atoms) in magenta, and slate blue, respectively. The lipid Phosphorus atom is shown as a orange sphere to represent the location of the membrane.

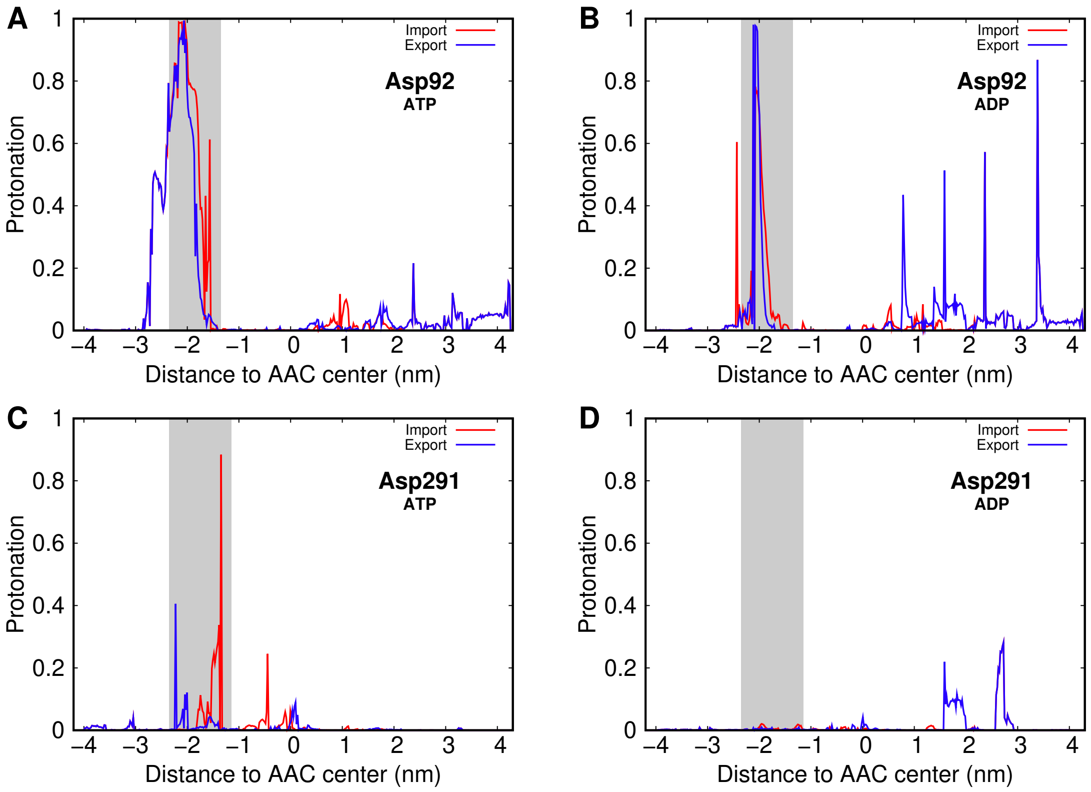

Figure S17: Protonation profiles of two acidic residues involved in the cytoplasmic salt-bridge network, Asp92 (A,B) and 291 (C,D), during the import (red line) and export (blue line) of both ATP (A,C) and ADP (B,D). A background grey area highlights the region sampled by each amino acid residue during the simulations, corresponding with the substrate interaction region. The spurious spikes that appear outside the grey area are most likely due to insufficient sampling coupled to the re-weight procedure.

References

(1) Williams, T.; Kelley, C.; many others, Gnuplot 4.6: an interactive plotting program. http://gnuplot.sourceforge.net/, 2013.

(2) Kabsch, W.; Sander, C. Dictionary of protein secondary structure: Pattern recognition of hydrogen-bonded and geometrical features. *Biopolymers* **1983**, *22*, 2577–2637.

(3) Touw, W. G.; Baakman, C.; Black, J.; te Beek, T. A.; Krieger, E.; Joosten, R. P.; Vriend, G. A series of PDB-related databanks for everyday needs. *Nucleic Acids Res.* **2014**, *43*, D364–D368.

(4) Schmid, N.; Eichenberger, A. P.; Choutko, A.; Riniker, S.; Winger, M.; Mark, A. E.; van Gunsteren, W. F. Definition and testing of the GROMOS force-field versions 54A7 and 54B7. *Eur. Biophys. J.* **2011**, *40*, 843.

(5) Oliveira, N. F. B.; Pires, I. D. S.; Machuqueiro, M. Improved GROMOS 54A7 Charge Sets for Phosphorylated Tyr, Ser, and Thr to Deal with pH-Dependent Binding Phenomena. *J. Chem. Theory Comput.* **2020**, *16*, 6368–6376.

(6) Thurlkill, R. L.; Grimsley, G. R.; Scholtz, J. M.; Ace, C. N. pK values of the ionizable groups of proteins. *Protein Sci.* **2006**, *15*, 1214–1218.

(7) Nury, H.; Dahout-Gonzalez, C.; Trezeguet, V.; Lauquin, G.; Brandolin, G.; ; Pebay-Peyroula, E. Relations between structure and function of the mitochondrial ADP/ATP carrier. *Annu. Rev. Biochem.* **2006**, *75*, 713–741.
